# Health-related quality of life in hereditary transthyretin amyloidosis polyneuropathy: a prospective, observational study

**DOI:** 10.1186/s13023-020-1340-x

**Published:** 2020-03-06

**Authors:** Mónica Inês, Teresa Coelho, Isabel Conceição, Lara Ferreira, Mamede de Carvalho, João Costa

**Affiliations:** 1 Instituto de Medicina Molecular, Av. Prof. Egas Moniz, 1649-028 Lisbon, Portugal; 2 Andrade’s Center for Familial Amyloidosis, Porto, Portugal; 3 Department of Neurosciences, Hospital de Santo António, Porto, Portugal; 4 Department of Neurosciences and Mental Health, Hospital de Santa Maria, Lisbon, Portugal; 5 University of the Algarve-ESGHT, Faro, Portugal; 6 Centre for Health Studies & Research, University of Coimbra, Coimbra, Portugal; 7 Laboratório de Farmacologia Clínica e Terapêutica, Faculdade de Medicina, Universidade de Lisboa, Lisbon, Portugal

**Keywords:** Amyloidosis, Hereditary transthyretin amyloid polyneuropathy, Health-related quality of life, Patient self-reported outcomes, hATTR-PN

## Abstract

**Background:**

Hereditary Transthyretin Amyloidosis Polyneuropathy is a rare life-threatening neurologic disease that imposes considerable mortality and it is associated with progressive related disabilities. In this study, we aimed to assess the effect of the disease across health-related quality of life dimensions, in both carriers of the mutation and patients, to compare health-related quality of life with general population, as well as to explore health-related quality of life prognostic factors among patients, including disease progression and treatment.

**Methods:**

This study was a multi-institutional, longitudinal, prospective, observational study of hereditary Transthyretin Amyloidosis Polyneuropathy Portuguese adult subjects (621 asymptomatic carriers and 733 symptomatic patients) enrolled in the Transthyretin Amyloidosis Outcomes Survey. Health-related quality of life was captured with the preference-based instrument EQ-5D-3 L. For general population the dataset included all subjects enrolled in a representative national study (*n* = 1500). Different econometric models were specified; multivariate probit, generalized linear model and generalized estimating equations model; including demographic and clinical covariates.

**Results:**

Hereditary Transthyretin Amyloidosis Polyneuropathy patients have their health status severely impaired in all quality of life dimensions and more anxiety/depression problems were found among asymptomatic carriers. No differences on utility were found between carriers and general population (*p* = 0.209). Among patients, the utility value is estimated to be 0.51 (0.021), a decrement of 0.27 as compared with general population utility. Higher disease duration, advanced disease stage and not receiving treatment are associated with impaired health-related quality of life. No differences were found between genders (*p* = 0.910) or between late (≥50 years) and early-onset patients (*p* = 0.254). The utility estimate ranged from 0.63 (0.009) in stage I to 0.01 (0.005) in stage IV.

**Conclusions:**

Hereditary Transthyretin Amyloidosis Polyneuropathy symptoms and progressive associated disabilities substantially decrease patient’s health-related quality of life. Clinical strategies focused on health-related quality of life preservation such as close follow-up of asymptomatic carriers, prompt diagnosis and adequate, early treatment would benefit patient’s long-term outcomes, slowing the progressive decline in health-related quality of life.

## Background

Hereditary transthyretin amyloid polyneuropathy (hATTR-PN) is a rare genetic disease, originally described in 1952 by Corino de Andrade in northern Portugal [1]. hATTR-PN is estimated to affect up to 10,000 people worldwide [2], the largest cohort of patient being Portuguese [3], almost all (> 99% families) carrying the Val30Met mutation [4]. The disease is related to mutations in the transthyretin (TTR) gene, leading to deposition of amyloid fibrils in the peripheral nerves and in vital organs [5]. Most Portuguese patients have their first symptoms between 28 and 42 years of age (interquartile range; median 33 years of age) and, if untreated, the disease progresses fast with the majority of patients dying within 12 years after disease onset [6]. Disease progression can be staged according to Coutinho approach [7]. Stage 1 is defined by symptoms primarily limited to the feet and legs, with pain and temperature sensation more severely impaired than touch, vibration, or position. Motor involvement is mild, with the patient being fully ambulatory. Autonomic dysfunction may be present and may be the presenting symptom, and can cause impotence, urinary retention, and gastrointestinal complications. In Stage 2 sensory impairment worsens and proceeds proximally, involving the upper limbs. In the lower limbs, touch sensation is lost. Motor dysfunction worsens, with the patient requiring assistance to walk with crutches or canes. Autonomic dysfunction can become more severe and more difficult to manage. In stage 3, the patient is profoundly affected by both the peripheral and autonomic impairments and is bedridden or confined to a wheelchair due to generalized weakness and severe cachexia [7].

Currently, two disease modifying treatment are available in clinical practise for patients in stage 1 (liver transplantation (LTx) since the 90’s [8–10] and tafamidis since 2011 [11, 12]) and two new pharmacologic treatments (inotersen and patisiran) were recently approved for the treatment of adult patients in stage 1 or stage 2 [13, 14].

Although there is significant clinical heterogeneity, even between patients carrying the same mutation, most subjects experience neuropathic pain and autonomic symptoms, such as gastrointestinal, urinary and erectile dysfunction, and orthostatic hypotension [15]. As the disease progresses, patients become severely disabled, malnourished, fatigued, weak, incontinent, bedridden or bound to a wheelchair, and unable to care for themselves [7, 16]. The activities of patients’ daily living became highly affected, leading to considerable emotional stress, loss of physical condition and independence, as well as to an increased need for familial, health care and social support [7, 15]. A recent study found neuropathy-specific quality of life among hATTR patients nearly equivalent to that of patients with type 2 diabetes with diabetic neuropathy accompanied by a history of ulceration, gangrene, or amputation [17]. Hence hATTR-PN imposes a considerable and increasing burden to patients, family and caregivers [18, 19].

In this study, we aimed to assess the effect of hATTR-PN across Health-Related Quality of Life (HRQoL) dimensions, in both carriers and patients, to estimate the impact of hATTR-PN on utility in comparison to the general population, as well as to explore HRQoL prognostic factors among patients, including disease progression and treatment.

## Methods

### Data

This study was a multi-institutional, longitudinal, observational study of hATTR-PN Portuguese adult subjects (≥18 years old) enrolled in the Transthyretin Amyloidosis Outcomes Survey (THAOS). The design and methodology of THAOS (ClinicalTrials.gov: NCT 00628745) are described in detail elsewhere [20, 21]. Briefly, THAOS is a prospective patient’s registry collecting demographic information, disease characteristics, current and prior treatments, family history, biopsy results and results of routine measurements performed in clinical practice, including the preference-based measure of health, EQ-5D-3L questionnaire.

In our study, the longitudinal follow-up of hATTR-PN patients was up to 10-years, since THAOS inception (2007) to study cut-off date (December 31, 2016). Patients were recruited at either one of the two Portuguese hATTR-PN referral centers. Data was extracted and validated at the referral centers, and discrepancies were resolved through revision of individual survey records. Both asymptomatic gene carriers and symptomatic patients were included. Non-Val30Met subjects and visits with incomplete EQ-5D-3L data were excluded from the analysis. For the general population, the dataset included all subjects enrolled in a representative adult Portuguese random sample, stratified by region, gender and age group. The detailed methods and results for the general population dataset have been described elsewhere [22].

HRQoL was captured with the preference-based instrument EQ-5D-3L. This instrument describes an individual’s HRQoL using a health classification system consisting of five dimensions: mobility, self-care, usual activities, pain/discomfort and anxiety/depression [23]. Each dimension has three associated severity levels, where level 1 represents the absence of health problems and level 3 represents extreme problems. The application of a value set to the perceived health state of the respondent generates the EQ-5D-3L index score (utility value) of the perceived health state [24]. The Portuguese value set yields an EQ-5D-3L index score that ranges from − 0.54 (for health states considered worse than death) to 1.00 (perfect health) [25]. For the general Portuguese population, EQ-5D data were collected in a cross-sectional study [22]. For hATTR-PN subjects, EQ-5D data were collected at THAOS enrolment (baseline) and in subsequent visits during clinical practice (longitudinal data). The Portuguese value set [25] was used to compute the EQ-5D-3L index score (utility value) for hATTR-PN subjects.

### Statistical analysis

Two analyses were conducted, making use of the two different available datasets. In the first analysis, cross-sectional data from the general population and from THAOS baseline visit (including both carriers and symptomatic patients) were pooled together, with the objective to assess the effect of the disease across quality of life dimensions and to estimate the disease impact on utility. In the second analysis, longitudinal data from symptomatic patients was used to explore possible prognostic factors associated with HRQoL. Pearson’s chi-square test was used to compare categorical variables between groups and Wilcoxon rank-sum test was used for comparisons of continuous variables. Crude utilities values (not based on models), standard errors (SE) and 95% confidence intervals (CI) were estimated.

In the first analysis, the independent variables included demographic covariates (age, sex and educational attainment) and two models were fitted to the cross-sectional data. First, for each EQ-5D dimension, a dependent binary variable was created to define the absence (severity level 1) or presence (severity level 2 or 3) of health problems. A 5-equation multivariate probit simultaneous model (model 1) was used to estimate several correlated binary outcomes jointly and to identify factors associated with the probability of reporting health problems among all EQ-5D dimensions. Second, the utility complement (disutility; defined as 1 − EQ-5D utility index score) was used as dependent variable in a model aiming to estimate the disease impact on utility (model 2). Different generalized linear models (GLM) were specified and the GLM with the best distribution and link function (as assessed through the Akaike and Bayesian information criteria) was used.

In the second analysis, the independent variables included demographic covariates, as well as clinical characteristics comprising age of onset (late (≥50 years) and early-onset patients), disease duration (defined as time since disease symptoms onset measured at baseline, i.e., at enrollment in THAOS or first EQ-5D response), treatment (untreated, treated with LTx or tafamidis) and disease stage. At study cut-off date there were no patients on other treatments participating on THAOS. Disease stage was captured in THAOS by the modified Polyneuropathy Disability (mPND) score that categorizes patients into five stages (I,II, IIIa, IIIb, IV) based on mobility status (walking without difficulty, walking with some difficulty, walking with 1 support, walking with 2 supports, not ambulatory) [26]. Patients with confirmed diagnosis, but lacking polyneuropathy symptoms were categorised as mPND stage I. The utility complement was used as dependent variable to fit a population-averaged panel-data (model 3) through a generalized estimating equations (GEE) model, accounting for the longitudinal features of patient’s dataset. To measure treatment effect adequately, patients with only one visit were excluded from this analysis. The GEE model with the best distribution and link function (as assessed through the Quasilikelihood under the Independence model) was used. We conducted a further analysis, using the same model, where mPND was mapped into Coutinho clinical stages [7] because Coutinho hATTR-PN stages is still the most common classification used in clinical practice. For this mapping, we used the following cut-offs: stage 1, if mPND ≤II or patients with confirmed diagnosis but lacking polyneuropathy symptoms; stage 2, if mPND = IIIa or IIIb; and stage 3, if mPND = IV. Additionally, an exploratory analysis was conducted to analyse possible differences in utility between the two Portuguese referral centers (Lisbon or Porto).

All statistical analysis was performed using Stata Statistical Software: Release 15.0 (StataCorp LLC, College Station, TX). The threshold for statistical significance was set at α = 0.05.

## Results

### Study sample

The general population dataset included 1500 Portuguese subjects. THAOS EQ-5D-3L data were available from 621 asymptomatic carriers and 733 symptomatic patients. A total of 4 non-Val30Met subjects and 5 subjects with incomplete EQ-5D-3L data were excluded from the analysis. Table 1 shows the main baseline characteristics of the three groups.

**Table 1:** Demographic characteristics

	General population	Asymptomatic Carriers^**a**^	Symptomatic Patients^**a**^
**No.**	1500	621	733
**Sex**			
Male, n (%)	711 (47.4)	218 (35.1)	378 (51.6)
**Age, years**			
Mean ± SD	48 ± 18.8	36.1 ± 13.3	42.7 ± 12.6
Median (IQR)	47 (32–64)	33 (26–44)	39 (34–49)
**Subjects by age group, n (%)**			
18–29	322 (21.7)	239 (38.5)	82 (11.2)
30–49	495 (33.3)	280 (45.1)	474 (64.7)
50–69	393 (26.5)	89 (14.3)	143 (19.5)
≥ 70	275 (18.5)	13 (2.1)	34 (4.6)
**Educational attainment, n (%)**			
Low	773 (51.9)	131 (21.3)	294 (40.4)
Medium (secondary)	401 (26.9)	323 (52.6)	321 (44.1)
High (bachelor, master or doctorate)	314 (21.1)	160 (26.1)	113 (15.5)

^a^Characteristics at enrollment or first EQ-5D response; SD denotes standard deviation; IQR denotes interquartile range

The proportion of male, age and educational attainment differed between groups (*p* < 0.001). As expected, median age was lower among asymptomatic carriers and patients, as the disease affects mainly young adulthood [3]. Asymptomatic carriers were mostly female, younger and with higher level of educational attainment, as compared with general population subjects and patients.

### EQ-5D dimensions

**Fig. 1:**
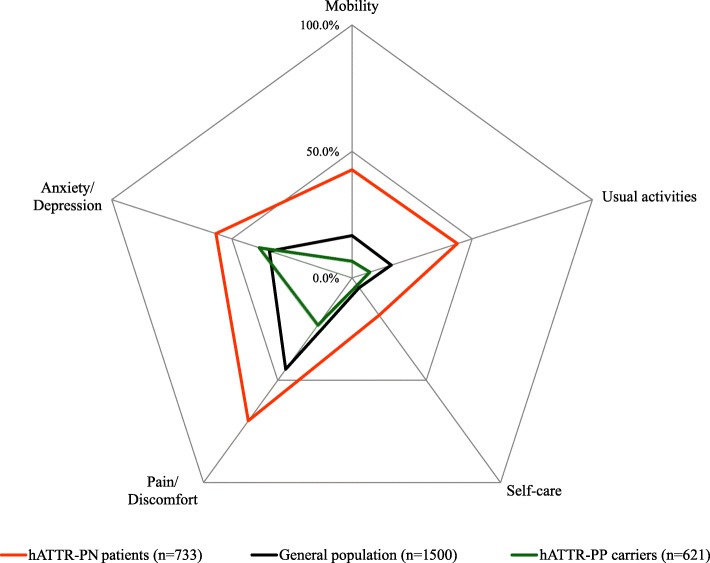
Proportion of subjects reporting health problems (severity level 2/3) by EQ-5D dimension

Figure 1 shows the crude distribution of health problems in the three groups as assessed with the EQ-5D. Among hATTR-PN patients, the EQ-5D dimensions more frequently compromised were pain/discomfort (70% of the patients), anxiety/depression (57% of the patients) and usual activities (44% of the patients) (Fig. 1). Among asymptomatic carriers, the EQ-5D dimension more frequently compromised was anxiety/depression (39% of the patients) (Fig. 1).

Model 1 estimated jointly the probability of reporting health problems for each EQ-5D dimension allowing for the fact that these probabilities might vary by group, but also due to other subject characteristics (e.g. sex, age). In comparison to the general population, hATTR-PN patients have their health status severely impaired in all five EuroQoL dimensions (*p* < 0.001). An additional table shows model coefficients in detail (see Additional file 1: Table 1). In comparison to the general population, asymptomatic carriers reported more frequently anxiety/depression problems, but less pain/discomfort problems (p < 0.001).

### Utilities

Model 2 estimated the utilities according to the group, while controlling for other subject characteristics (e.g. sex, age). The model estimates utility scores with reference to a base-case subject profile (the reference case is general population, female, 18–29 years of age with low educational attainment). Older age, female sex, lower educational attainment and symptomatic disease were associated with poorer HRQoL (*p* < 0.001) (see Additional file 1: Table 2). Overall, no differences in utility were found between carriers and the general population (*p* = 0.209). The utility value for a hATTR-PN patient was estimated to be 0.51 (SE 0.021), a decrement of 0.27 versus the general population (0.78, SE 0.006). All sex and age groups of hATTR-PN patients showed a utility decrement (Table 2).

**Table 2:** Mean utility (SE) in each group, by sex and age group

	General Population		Carriers		Patients	
Age group	Female	Male	Female	Male	Female	Male
18–29	0.84 (0.014)	0.88 (0.012)	0.86 (0.008)	0.88 (0.007)	0.65 (0.022)	0.71 (0.018)
30–49	0.81 (0.014)	0.84 (0.015)	0.82 (0.009)	0.85 (0.008)	0.56 (0.021)	0.63 (0.016)
50–69	0.65 (0.017)	0.74 (0.018)	0.72 (0.017)	0.76 (0.015)	0.31 (0.041)	0.43 (0.033)
≥70	0.56 (0.025)	0.66 (0.025)	0.64 (0.027)	0.70 (0.024)	0.13 (0.065)	0.28 (0.054)
All	0.78 (0.006)		0.80 (0.009)		0.51 (0.021)	

Model 3 estimated the utilities for health states (mPND stage) among hATTR-PN patients, while exploring the influence of demographic and other clinical covariates, and an interaction between visits and treatment. Table 3 shows the clinical characteristics of hATTR-PN patients analyzed in the model.

**Table 3:** hATTR-PN patients clinical characteristics

Characteristics	Patients
**No. Subjects (no. visits)**	733 (2913)
**Age at onset, years**	
Mean ± SD	38.1 ± 12.6
Median (IQR)	34 (29–44)
**Late-onset, n (%)**	116 (15.8)
**Disease duration at THAOS enrolment, years**	
Mean ± SD	4.5 ± 4.8
**Age at diagnosis, years**	
Mean ± SD	39.4 ± 12.9
Median (IQR)	35 (30–45)
**Disease mPND stage, n (%)**	
stage I	537 (73.3)
stage II	141 (19.2)
stage IIIa	22 (3)
stage IIIb	12 (1.6)
stage IV	21 (2.9)
**Disease Coutinho stage, n (%)**	
stage 1	678 (92.5)
stage 2	34 (4.6)
stage 3	21 (2.9)
**Visit, n**	
1	733
2	641
3	526
4	419
5	298
6	187
7	88
8	16
9	5
**Treated, n (%)**	637 (86.9)

Clinical characteristics measured at THAOS enrolment or first EQ-5D-3L response; Treatment variable status measured across follow-up; SD denotes standard deviation, IQR interquartile range

Older age (≥70 years), lower educational attainment, higher disease duration, not receiving treatment and higher mPND disease stage were associated with poorer HRQoL (*p* < 0.05). Overall expected utility values for untreated patients and treated patients was 0.56 (0.012) and 0.59 (0.009), respectively. In general, a decline in quality of life was observed among untreated patients over time (visits) (*p* < 0.05), while it was found that treated patients increased/preserved their quality of life through the years (interaction term treatment/visit, p < 0.05) (see Additional file 1: Figure 1). No differences in utility was found between genders (*p* = 0.910) or among early patients as compared with those with late-onset disease (*p* = 0.254). An additional table shows model coefficients in detail (see Additional file 1: Table 3). Results of the exploratory analysis found no difference in utility between referral centers (*p* = 0.210).

**Fig. 2:**
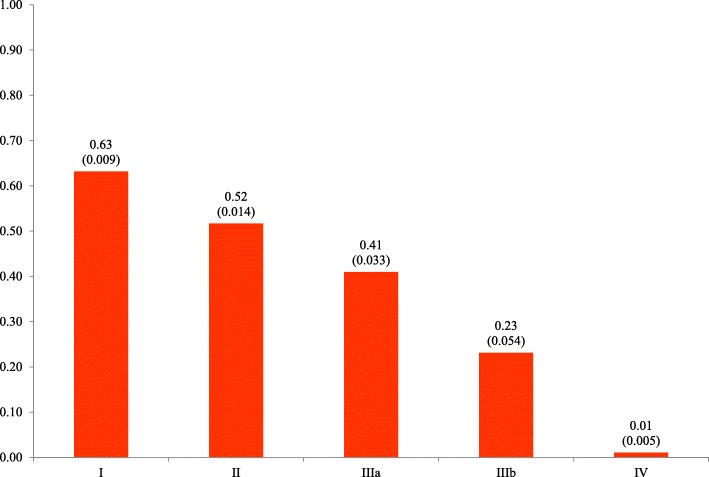
Mean utility (SE), by hATTR-PN mPND disease stage

**Fig. 3:**
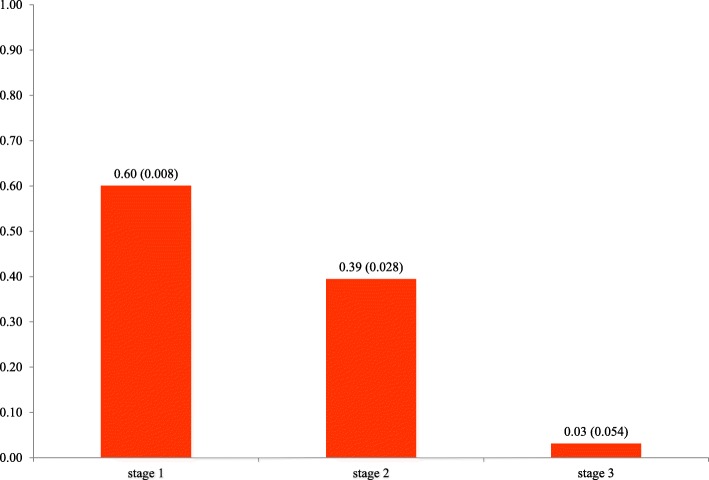
Mean utility (SE), by hTTR-PN Coutinho clinical stage

Figure 2 shows the expected utility values for each disease mPND stage, documenting a substantial impairment in HRQoL as disease progresses. Similar results were found when disease progression is captured by Coutinho stages (Fig. 3).

An additional table shows model coefficients in detail (see Additional file 1: Table 4).

## Discussion

Disease rarity can cause uncertainty in estimates [27] that may reveal to be determinant in health technology assessment [28–30], such as utility values. In this study, we aimed to assess the effect of hATTR-PN across HRQoL dimensions, in both carriers and patients, to estimate the impact on utility in comparison to the general population, as well as to explore HRQoL prognostic factors among patients, including disease progression (clinical stage) and treatment.

The main findings of our study are as follows. First, a high proportion of hATTR-PN patients have some impairment on each of these HRQoL dimensions: mobility, self-care, usual activities, pain/discomfort and anxiety/depression. Second, although average utility was not different between asymptomatic carriers and the general population, the probability of carriers experiencing anxiety/depression problems is higher, which is consistent with the published literature on the psychological consequences of pre-symptomatic genetic testing such as anxiety, depression, avoidance/denial of the disease, and psychological distress [31–34]. This result reinforces the impact that emotional and psychological factors may have, not only among patients but also among carriers of the disease-causing mutation.

Third, interestingly, carriers reported less pain/discomfort problems in comparison to the general population, which can be due to a misclassification bias as a result from inaccurate recall. Although the reasons to explain this finding remain unclear, possible explanations may be associated with the carrier’s knowledge from living with relatives with the disease (which experience pain/discomfort) that may prompt carriers to report less likely these problems and with carrier’s disease denial. Fourth, average utility among hATTR-PN patients is about two-thirds of the general population, a difference higher than values often used to establish a minimally important difference [35, 36]. This clinically important difference is observed across all sex and age groups. As compared with other chronic diseases patients, hATTR-PN utility (0.51) is lower than utilities for psoriasis (0.75) [37], type 2 diabetes mellitus (0.68) [38], heart failure (0.63) [39] and rheumatoid arthritis (0.62) [40], although higher than for visual impairment condition (0.44) [41]. Fifth, older age, lower educational attainment, higher disease duration, not receiving treatment and higher disease stage were found to be determinants of poorer HRQoL among hATTR-PN patients, without differences between genders or between early and late-onset patients. The HRQoL among untreated patients was found deteriorating over time, while treated patients increased and preserved their HRQoL through the years. Average utility of late-stage hATTR-PN patient is similar to the value assumed for the death health state.

Previous studies evaluating HRQoL on patients with hATTR-PN either excluded patients who were already non ambulatory or had advanced neurological disability [17], enrolled a small number of patients at advanced disease stages (*n* ≤ 15) [42], lacked a comprehensive analysis of HRQoL clinical prognostic determinants [20], or excluded LTx-treated patients [43]. Furthermore, other methodological aspects from these previous studies may be debatable, such as applying a specific country EQ-5D value set to preference-based questionnaires provided by subjects from different geographies [20]. Nevertheless, our results are in agreement with these previous studies, showing HRQoL impairment among hATTR-PN patients, but not among carriers, in comparison to the general population [20], a very low utility value at latter stages of the disease [42], and a positive treatment effect on HRQoL patients [43].

The main strength of our patient reported outcome (PRO) study is the inclusion of over 1300 hATTR-PN Val30Met patients and asymptomatic carriers, which were followed prospectively. In addition to provide a comprehensive analysis of HRQoL among hATTR-PN patients and carriers, we anticipate that our results are relevant for the process of health economics technology assessment. Cost-effectiveness decision analysis usually requires preference-based HRQoL data (utility values) to estimate quality-adjusted life-years (QALY). This is of particular importance for hATTR-PN, because two new drugs (inotersen and patisiran) have recently received market authorization in Europe and US for hATTR-PN [13, 14]. Applying country tariffs to raw EQ-5D response data from a large longitudinal registry is preferred by HTA agencies [44] but difficulties remain due to disease rarity. One of the most challenging decisions that payers face when adopting an orphan drug is related to the uncertainty of the HRQoL estimates due to small sample and poor-quality data.

Our study has some limitations. First, although the sample showed good variation in demographic and clinical characteristics, it may have been subject to selection bias due to the observational nature of the study. The THAOS sample here analyzed represents nearly 57% of Portuguese hATTR-PN patients followed at the referral centers during 2016. Nevertheless, this sample was younger and included more men [6]. Furthermore, our sample may underrepresent the population of patients at later disease stages, as those are usually wheelchair/bedridden and less willing and less likely to participate in prospective registries. Moreover, small sample sizes in the later stages can limit the precision of estimated coefficients for stage differences. Second, all hATTR-PN patients and carriers had the Val30Met mutation. Although having the same mutation warrant genetic homogeneity to our sample, caution is needed in generalize the results to populations with other TTR mutations and disease presentations, which are more common in other geographies [45]. Third, utility scores rely on EQ-5D-3L, a patient self-reported outcome instrument. Errors inherent to self-report can seriously bias the estimates. PRO measures play a key role in patient-centered care research but the quality of results and the true nature of the associations depend on the validity of the measures [46]. In this study, demographic and clinical characteristics were verified by the referral centers which increase overall data validity. Fourth, for the comparison of HRQoL between the three groups, we were only able to use cross-sectional data. Prospective data was only used for exploring HRQoL prognostic factors among patients. Nevertheless, the results found across groups strongly suggest that EQ-5D-3L is a valid instrument in assessing HRQoL among hATTR-PN patients.

## Conclusions

Due to the small number of people with rare diseases, there is more uncertainty about the health gains from treatments and the best way to decrease this uncertainty is to collect long-term outcomes data, through large patient’s registries such as THAOS. In the case of hATTR-PN patients, we can conclude that clinical strategies focused on quality of life preservation such as close follow-up of asymptomatic carriers, prompt diagnosis and adequate and early treatment would largely benefit patient’s long-term outcomes.

## Supplementary information

**Additional file 1.** Supplemental material. Description of data: Econometric outputs of model 1, 2, 3 and 4 and predictive margins of treatment, across visits/years.

## Data Availability

The hATTR-PN dataset is part of THAOS (sharing statement https://clinicaltrials.gov/ct2/show/NCT00628745?term=THAOS&rank=1). The Portuguese general population dataset is available on reasonable request from individuals affiliated with research or healthcare institutions located in Portugal, as approved by the Portuguese National Data Protection Committee.
