# Supplementary material for: Health-related quality of life in hereditary transthyretin amyloidosis polyneuropathy: a prospective, observational study
Source: Orphanet J Rare Dis. 2020 Mar 6;15:67. doi: 10.1186/s13023-020-1340-x (PMC7060628; doi:10.1186/s13023-020-1340-x)
Supplement: Supplementary file 1 — Additional file 1. Supplemental material. Description of data: Econometric outputs of model 1, 2, 3 and 4 and predictive margins of treatment, across visits/years. [file 13023_2020_1340_MOESM1_ESM.docx]

**ADDITIONAL FILE 1**

**Table 1. Econometric outputs of model 1 (multivariate probit model) for probability of reporting health problems (level of 2 or 3 in EQ-5D dimensions).**

| EQ-5D dimension/Parameter | Coefficient | SE | z | P>z | [95% CI] | |
| --- | --- | --- | --- | --- | --- | --- |
| Mobility |  |  |  |  |  |  |
| Group |  | | | | | |
| general population | (reference) | | | | | |
| hTTR-PN asymptomatic carrier | -0.18 | 0.1 | -1.8 | 0.072 | -0.376 | 0.016 |
| hTTR-PN symptomatic patient | 1.096 | 0.072 | 15.14 | 0.000 | 0.954 | 1.238 |
| Sex |  | | | | | |
| female | (reference) | | | | | |
| male | -0.051 | 0.06 | -0.85 | 0.394 | -0.17 | 0.067 |
| Age group |  | | | | | |
| 18–29 | (reference) | | | | | |
| 30–49 | 0.463 | 0.107 | 4.32 | 0.000 | 0.253 | 0.673 |
| 50–69 | 1.113 | 0.115 | 9.7 | 0.000 | 0.888 | 1.338 |
| ≥70 | 1.432 | 0.128 | 11.17 | 0.000 | 1.18 | 1.683 |
| Educational attainment |  | | | | | |
| Low | (reference) | | | | | |
| Medium (secondary) | -0.324 | 0.074 | -4.39 | 0.000 | -0.469 | -0.179 |
| High (bachelor, master or doctorate) | -0.605 | 0.092 | -6.59 | 0.000 | -0.785 | -0.425 |
| Intercept | -1.624 | 0.116 | -13.99 | 0.000 | -1.852 | -1.397 |
| Usual activities |  |  |  |  |  |  |
| Group |  | | | | | |
| general population | (reference) | | | | | |
| hTTR-PN asymptomatic carrier | -0.167 | 0.092 | -1.82 | 0.069 | -0.347 | 0.013 |
| hTTR-PN symptomatic patient | 1.092 | 0.069 | 15.76 | 0.000 | 0.956 | 1.227 |
| Sex |  | | | | | |
| female | (reference) | | | | | |
| male | -0.197 | 0.058 | -3.37 | 0.001 | -0.312 | -0.083 |
| Age group |  | | | | | |
| 18–29 | (reference) | | | | | |
| 30–49 | 0.341 | 0.094 | 3.64 | 0.000 | 0.158 | 0.525 |
| 50–69 | 0.853 | 0.103 | 8.29 | 0.000 | 0.652 | 1.055 |
| ≥70 | 1.164 | 0.117 | 9.92 | 0.000 | 0.934 | 1.394 |
| Educational attainment |  | | | | | |
| Low | (reference) | | | | | |
| Medium (secondary) | -0.284 | 0.071 | -3.98 | 0.000 | -0.424 | -0.145 |
| High (bachelor, master or doctorate) | -0.487 | 0.085 | -5.72 | 0.000 | -0.654 | -0.32 |
| Intercept | -1.391 | 0.103 | -13.48 | 0.000 | -1.594 | -1.189 |
| Self-care |  |  |  |  |  |  |
| Group |  | | | | | |
| general population | (reference) | | | | | |
| hTTR-PN asymptomatic carrier | 0.222 | 0.121 | 1.83 | 0.067 | -0.016 | 0.46 |
| hTTR-PN symptomatic patient | 1.032 | 0.089 | 11.57 | 0.000 | 0.858 | 1.207 |
| Sex |  | | | | | |
| female | (reference) | | | | | |
| male | 0.085 | 0.075 | 1.13 | 0.259 | -0.062 | 0.232 |
| Age group |  | | | | | |
| 18–29 | (reference) | | | | | |
| 30–49 | 0.239 | 0.148 | 1.61 | 0.107 | -0.051 | 0.53 |
| 50–69 | 0.908 | 0.155 | 5.86 | 0.000 | 0.604 | 1.211 |
| ≥70 | 1.048 | 0.17 | 6.16 | 0.000 | 0.714 | 1.381 |
| Educational attainment |  | | | | | |
| Low | (reference) | | | | | |
| Medium (secondary) | -0.402 | 0.095 | -4.25 | 0.000 | -0.588 | -0.217 |
| High (bachelor, master or doctorate) | -0.663 | 0.137 | -4.85 | 0.000 | -0.931 | -0.395 |
| Intercept | -2.203 | 0.162 | -13.62 | 0.000 | -2.52 | -1.886 |
| Pain/Discomfort |  |  |  |  |  |  |
| Group |  | | | | | |
| general population | (reference) | | | | | |
| hTTR-PN asymptomatic carrier | -0.369 | 0.07 | -5.31 | 0.000 | -0.506 | -0.233 |
| hTTR-PN symptomatic patient | 0.867 | 0.064 | 13.63 | 0.000 | 0.742 | 0.992 |
| Sex |  | | | | | |
| female | (reference) | | | | | |
| male | -0.284 | 0.051 | -5.55 | 0.000 | -0.384 | -0.184 |
| Age group |  | | | | | |
| 18–29 | (reference) | | | | | |
| 30–49 | 0.197 | 0.068 | 2.88 | 0.004 | 0.063 | 0.332 |
| 50–69 | 0.796 | 0.08 | 9.94 | 0.000 | 0.639 | 0.953 |
| ≥70 | 1.096 | 0.101 | 10.87 | 0.000 | 0.899 | 1.294 |
| Educational attainment |  | | | | | |
| Low | (reference) | | | | | |
| Medium (secondary) | -0.171 | 0.062 | -2.74 | 0.006 | -0.293 | -0.048 |
| High (bachelor, master or doctorate) | -0.369 | 0.07 | -5.24 | 0.000 | -0.506 | -0.231 |
| Intercept | -0.363 | 0.078 | -4.65 | 0.000 | -0.517 | -0.21 |
| Anxiety/Depression |  |  |  |  |  |  |
| Group |  | | | | | |
| general population | (reference) | | | | | |
| hTTR-PN asymptomatic carrier | 0.23 | 0.065 | 3.52 | 0.000 | 0.102 | 0.358 |
| hTTR-PN symptomatic patient | 0.654 | 0.061 | 10.66 | 0.000 | 0.534 | 0.775 |
| Sex |  | | | | | |
| female | (reference) | | | | | |
| male | -0.274 | 0.049 | -5.54 | 0.000 | -0.37 | -0.177 |
| Age group |  | | | | | |
| 18–29 | (reference) | | | | | |
| 30–49 | 0.05 | 0.066 | 0.77 | 0.444 | -0.078 | 0.179 |
| 50–69 | 0.342 | 0.078 | 4.38 | 0.000 | 0.189 | 0.495 |
| ≥70 | 0.175 | 0.097 | 1.8 | 0.072 | -0.015 | 0.365 |
| Educational attainment |  | | | | | |
| Low | (reference) | | | | | |
| Medium (secondary) | -0.256 | 0.061 | -4.18 | 0.000 | -0.376 | -0.136 |
| High (bachelor, master or doctorate) | -0.296 | 0.069 | -4.3 | 0.000 | -0.431 | -0.161 |
| Intercept | -0.291 | 0.076 | -3.83 | 0.000 | -0.441 | -0.142 |
| SE - standard error; CI - Confidence Interval. Note: To calculate the estimated probabilities, the coefficients should be sum up and them applied the cumulative distribution function of the standard normal distribution. | | | | | | |

**Table 2. Econometric outputs of model 2 (generalized linear model; gamma distribution, log link) for utility complement.**

| Parameter | Coefficient | SE | z | P>z | [95% CI] | |
| --- | --- | --- | --- | --- | --- | --- |
| Group |  | | | | | |
| general population | (reference) | | | | | |
| hTTR-PN asymptomatic carrier | -0.065 | 0.052 | -1.26 | 0.209 | -0.166 | 0.036 |
| hTTR-PN symptomatic patient | 0.823 | 0.049 | 16.72 | 0.000 | 0.727 | 0.92 |
| Sex |  | | | | | |
| female | (reference) | | | | | |
| male | -0.184 | 0.039 | -4.67 | 0.000 | -0.261 | -0.107 |
| Age group |  | | | | | |
| 18–29 | (reference) | | | | | |
| 30–49 | 0.226 | 0.052 | 4.38 | 0.000 | 0.125 | 0.328 |
| 50–69 | 0.674 | 0.062 | 10.89 | 0.000 | 0.553 | 0.795 |
| ≥70 | 0.907 | 0.076 | 11.93 | 0.000 | 0.758 | 1.056 |
| Educational attainment |  | | | | | |
| Low | (reference) | | | | | |
| Medium (secondary) | -0.292 | 0.048 | -6.03 | 0.000 | -0.386 | -0.197 |
| High (bachelor, master or doctorate) | -0.416 | 0.054 | -7.64 | 0.000 | -0.522 | -0.309 |
| Intercept | -1.691 | 0.059 | -28.42 | 0.000 | -1.808 | -1.574 |
| SE - standard error; CI - Confidence Interval. Note: To calculate the utility values, the coefficients should be sum up, applied the exponential function and then deducted from “1”. | | | | | | |

**Table 3. Econometric outputs of model 3 (generalized estimating equations; gaussian distribution, identity link) for hTTR-PN patients’ utility complement (mPND stages).**

| Parameter | Coefficient | SE | z | P>z | [95% CI] | |
| --- | --- | --- | --- | --- | --- | --- |
| Sex |  | | | | | |
| female | (reference) | |  |  |  |  |
| male | -0.021 | 0.017 | -1.27 | 0.205 | -0.054 | 0.012 |
| Age group |  |  |  |  |  |  |
| 18–29 | (reference) | |  |  |  |  |
| 30–49 | -0.005 | 0.027 | -0.17 | 0.865 | -0.058 | 0.049 |
| 50–69 | 0.055 | 0.046 | 1.20 | 0.231 | -0.035 | 0.146 |
| ≥70 | 0.133 | 0.066 | 2.01 | 0.044 | 0.003 | 0.263 |
| Educational attainment |  |  |  |  |  |  |
| Low | (reference) | |  |  |  |  |
| Medium (secondary) | -0.062 | 0.020 | -3.09 | 0.002 | -0.101 | -0.023 |
| High (bachelor, master or doctorate) | -0.097 | 0.025 | -3.80 | 0.000 | -0.147 | -0.047 |
| Disease duration, years | 0.007 | 0.002 | 3.37 | 0.001 | 0.003 | 0.011 |
| Disease onset |  |  |  |  |  |  |
| Early | (reference) | |  |  |  |  |
| Late | 0.074 | 0.044 | 1.69 | 0.092 | -0.012 | 0.160 |
| mPND stage |  |  |  |  |  |  |
| I | (reference) | |  |  |  |  |
| II | 0.115 | 0.015 | 7.46 | 0.000 | 0.085 | 0.145 |
| IIIa | 0.222 | 0.034 | 6.54 | 0.000 | 0.156 | 0.289 |
| IIIb | 0.400 | 0.055 | 7.24 | 0.000 | 0.292 | 0.509 |
| IV | 0.621 | 0.056 | 11.02 | 0.000 | 0.511 | 0.731 |
| Treatment |  |  |  |  |  |  |
| Untreated | (reference) | |  |  |  |  |
| Treated | 0.035 | 0.016 | 2.26 | 0.024 | 0.005 | 0.066 |
| Visit |  |  |  |  |  |  |
| 1 | (reference) | |  |  |  |  |
| 2 | 0.029 | 0.014 | 2.05 | 0.040 | 0.001 | 0.057 |
| 3 | 0.029 | 0.019 | 1.51 | 0.131 | -0.009 | 0.066 |
| 4 | 0.050 | 0.025 | 1.96 | 0.050 | 0.000 | 0.100 |
| 5 | 0.035 | 0.033 | 1.05 | 0.293 | -0.030 | 0.099 |
| 6 | 0.120 | 0.037 | 3.28 | 0.001 | 0.048 | 0.192 |
| 7 | 0.166 | 0.055 | 2.99 | 0.003 | 0.057 | 0.275 |
| 8 | 0.203 | 0.162 | 1.25 | 0.211 | -0.115 | 0.521 |
| 9 | 0.203 | 0.162 | 1.25 | 0.211 | -0.115 | 0.521 |
| Interaction Treatment/Visit |  |  |  |  |  |  |
| Treated/Visit 2 | -0.052 | 0.020 | -2.59 | 0.010 | -0.091 | -0.013 |
| Treated/Visit 3 | -0.062 | 0.024 | -2.60 | 0.009 | -0.109 | -0.015 |
| Treated/Visit 4 | -0.089 | 0.030 | -3.01 | 0.003 | -0.147 | -0.031 |
| Treated/Visit 5 | -0.077 | 0.037 | -2.10 | 0.036 | -0.149 | -0.005 |
| Treated/Visit 6 | -0.170 | 0.041 | -4.15 | 0.000 | -0.250 | -0.090 |
| Treated/Visit 7 | -0.202 | 0.060 | -3.36 | 0.001 | -0.320 | -0.084 |
| Treated/Visit 8 | -0.228 | 0.169 | -1.35 | 0.178 | -0.559 | 0.104 |
| Treated/Visit 9 | -0.221 | 0.182 | -1.21 | 0.225 | -0.577 | 0.136 |
| Intercept | 0.364 | 0.033 | 11.14 | 0.000 | 0.300 | 0.428 |
| SE - standard error; CI - Confidence Interval. Note: To calculate the utility values, the coefficients should be sum up and then deducted from “1”. | | | | | | |

**Figure 1.** **Predictive margins of treatment, by group (0=untreated; 1=treated) across visits/years.**

Note: Caution should be taken interpreting estimates for later visits (8-9) as the number of patients with more than 7 visits is small which can limit the precision of estimated coefficients for visit differences.

**Table 4. Econometric outputs of model 3 (generalized estimating equations; gaussian distribution, identity link) for hTTR-PN patients’ utility complement (Coutinho clinical polyneuropathy stages).**

| Parameter | Coefficient | SE | z | P>z | [95% CI] | |
| --- | --- | --- | --- | --- | --- | --- |
| Sex |  | | | | | |
| female | (reference) | |  |  |  |  |
| male | -0.002 | 0.017 | -0.11 | 0.910 | -0.034 | 0.031 |
| Age group |  |  |  |  |  |  |
| 18–29 | (reference) |  |  |  |  |  |
| 30–49 | 0.009 | 0.027 | 0.33 | 0.739 | -0.045 | 0.063 |
| 50–69 | 0.106 | 0.046 | 2.31 | 0.021 | 0.016 | 0.196 |
| ≥70 | 0.204 | 0.064 | 3.2 | 0.001 | 0.079 | 0.329 |
| Educational attainment |  |  |  |  |  |  |
| Low | (reference) |  |  |  |  |  |
| Medium (secondary) | -0.063 | 0.02 | -3.17 | 0.002 | -0.102 | -0.024 |
| High (bachelor, master or doctorate) | -0.109 | 0.025 | -4.27 | 0.000 | -0.159 | -0.059 |
| Disease duration, years | 0.009 | 0.002 | 4.22 | 0.000 | 0.005 | 0.013 |
| Disease onset |  |  |  |  |  |  |
| Early | (reference) |  |  |  |  |  |
| Late | 0.05 | 0.044 | 1.14 | 0.254 | -0.036 | 0.135 |
| Disease stage |  |  |  |  |  |  |
| stage 1 | (reference) |  |  |  |  |  |
| stage 2 | 0.206 | 0.029 | 7.16 | 0.000 | 0.149 | 0.262 |
| stage 3 | 0.569 | 0.054 | 10.5 | 0.000 | 0.463 | 0.675 |
| Treatment |  |  |  |  |  |  |
| Untreated | (reference) |  |  |  |  |  |
| Treated | 0.031 | 0.015 | 2.04 | 0.042 | 0.001 | 0.061 |
| Visit |  |  |  |  |  |  |
| 1 | (reference) |  |  |  |  |  |
| 2 | 0.03 | 0.014 | 2.15 | 0.032 | 0.003 | 0.058 |
| 3 | 0.033 | 0.019 | 1.75 | 0.081 | -0.004 | 0.07 |
| 4 | 0.055 | 0.025 | 2.17 | 0.030 | 0.005 | 0.105 |
| 5 | 0.045 | 0.033 | 1.38 | 0.167 | -0.019 | 0.11 |
| 6 | 0.145 | 0.036 | 3.97 | 0.000 | 0.073 | 0.216 |
| 7 | 0.202 | 0.055 | 3.65 | 0.000 | 0.093 | 0.31 |
| 8 | 0.211 | 0.162 | 1.3 | 0.195 | -0.108 | 0.529 |
| 9 | 0.211 | 0.162 | 1.3 | 0.195 | -0.108 | 0.529 |
| Interaction Treatment/Visit |  |  |  |  |  |  |
| Treated/Visit 2 | -0.047 | 0.02 | -2.38 | 0.017 | -0.086 | -0.008 |
| Treated/Visit 3 | -0.059 | 0.024 | -2.5 | 0.013 | -0.106 | -0.013 |
| Treated/Visit 4 | -0.085 | 0.029 | -2.89 | 0.004 | -0.143 | -0.027 |
| Treated/Visit 5 | -0.076 | 0.037 | -2.06 | 0.039 | -0.147 | -0.004 |
| Treated/Visit 6 | -0.183 | 0.041 | -4.49 | 0.000 | -0.263 | -0.103 |
| Treated/Visit 7 | -0.225 | 0.06 | -3.75 | 0.000 | -0.342 | -0.107 |
| Treated/Visit 8 | -0.218 | 0.169 | -1.29 | 0.198 | -0.549 | 0.114 |
| Treated/Visit 9 | -0.22 | 0.182 | -1.21 | 0.227 | -0.576 | 0.137 |
| Intercept | 0.362 | 0.033 | 11.07 | 0.000 | 0.298 | 0.426 |
| SE - Standard Error; CI - Confidence Interval. Note: To calculate the utility values, the coefficients should be sum up and then deducted from “1”. | | | | | | |
